# Comparative Genomic and Transcriptomic Analyses of Family-1 UDP Glycosyltransferase in Prunus Mume

**DOI:** 10.3390/ijms19113382

**Published:** 2018-10-29

**Authors:** Zhiyong Zhang, Xiaokang Zhuo, Xiaolan Yan, Qixiang Zhang

**Affiliations:** 1 Beijing Key Laboratory of Ornamental Plants Germplasm Innovation & Molecular Breeding, National Engineering Research Center for Floriculture, Beijing Laboratory of Urban and Rural Ecological Environment, Engineering Research Center of Landscape Environment of Ministry of Education, Key Laboratory of Genetics and Breeding in Forest Trees and Ornamental Plants of Ministry of Education, School of Landscape Architecture, Beijing Forestry University, Beijing 100083, China; zhangzhiyong5432@gmail.com (Z.Z.); zhuoxk@bjfu.edu.cn (X.Z.); 2 Beijing Advanced Innovation Center for Tree Breeding by Molecular Design, Beijing Forestry University, Beijing 100083, China; 3 Mei Flower Research Center in China, Wuhan 430074, China; zhangzhiyongapply@gmail.com

**Keywords:** *Prunus mume*, UGT family, evolutionary divergence, expression analysis, hormone

## Abstract

Glycosylation mediated by Family-1 UDP-glycosyltransferases (UGTs) plays crucial roles in plant growth and adaptation to various stress conditions. *Prunus mume* is an ideal crop for analyzing flowering for its early spring flowering characteristics. Revealing the genomic and transcriptomic portfolio of the UGT family in *P. mume*, a species in which *UGTs* have not yet been investigated, is therefore important. In this study, 130 putative *UGT* genes were identified and phylogenetically clustered into 14 groups. These *PmUGTs* were distributed unevenly across eight chromosomes and 32 tandem duplication and 8 segmental duplication pairs were revealed. A highly conserved intron insertion event was revealed on the basis of intron/exon patterns within *PmUGTs*. According to RNA-seq data, these *PmUGTs* were specifically expressed in different tissues and during the bud dormancy process. In addition, we confirmed the differential expression of some representative genes in response to abscisic acid treatment. Our results will provide important information on the UGT family in *P. mume* that should aid further characterization of their biological roles in response to environmental stress.

## 1. Introduction

Increasing evidence is suggesting that glycosylation mediated by glycosyltransferases (GTs) plays crucial roles in plant growth and response to biotic and abiotic stresses [1]. According to numerous studies, GTs catalyze the transfer of sugar moieties from active sugar molecules to a variety of acceptor molecules, namely, hormones, lipids and some other small molecules [1,2]. The formation of a glycosidic bond can change an acceptor’s chemical properties and bioactivity, adjustments that are essential for the maintenance of cellular homeostasis. In addition, conjugation by GTs allows plant cells to modulate their biochemical proprieties and thus have a strong influence on their biological activity and compartmental storage [3].

GTs constitute a highly diverse, multigene family [4]. To date, 105 GT families have been identified in the carbohydrate-active enzyme database (CAZy, available online: http://www.cazy.org/) the largest of which is family 1 (GT1) [5,6]. Because it uses UDP-glucose as the sugar donor molecule, GT1 is also known as UDP-glycosyltransferase (UGT) [1]. UGTs possess a highly conserved 44-amino-acid C-terminal consensus sequence, referred to as the plant secondary product glycosyltransferase (PSPG) box [7,8]. Putative UGT genes have recently been identified in many plants, including 107 in *Arabidopsis*, 148 in *Glycine max* and 148 in *Zea mays* [4,9,10,11]. In perennial trees, the number of isolated putative UGT genes includes 168 in *Prunus persica*, 254 in *Malus domestica* and 184 in *Vitis vinifera* [12,13]*.*

Phytohormones have been thoroughly demonstrated to play critical roles in developmental processes and to adapt to external environmental changes [14,15,16,17,18]. Plants have therefore evolved a range of mechanisms to keep different hormones in homeostasis [19]. Glycosylation is thought to be one of these mechanisms. Abscisic acid (ABA) is a relatively well-studied phytohormone that is critical for plant development. To adapt to changing environmental conditions, plants must fine-tune ABA levels and keep different ABA forms in balance [20]. The conjugation of ABA with ABA-glucose ester (ABA-GE) is a well-studied phenomenon that changes ABA bioactivity. Several ABA-related *UGTs* have been functionally characterized, such as *UGT71B6* (*Arabidopsis*), *ABAGT* (*V. angularis*) and *UGT71A35* (strawberry) [21,22,23]. In regard to indole-3-acetic acid (IAA), the first identified *UGT* was *IAGLU* in maize [24]. In *Arabidopsis*, IAA-related UGT (*UGT84B1*) has also been recently isolated and its overexpression leads to an auxin deficiency phenotype [25]. Overexpression of *UGT73C5*, another *UGT* of *Arabidopsis*, reduces levels of active brassinosteroid (BR), with transgenic plants displaying BR-deficient phenotypes, which suggests that *UGT73C5* glucosylates BR and reduces its bioactivity [26]. *UGT76C1* and *UGT76C2*, two *UGTs* with *N*-glucosyltransferase activity toward cytokinins, have also been identified [27]. Two other *UGTs*, *UGT74F*1 and *UGT74F2*, are active toward salicylic acid (SA) and benzoic acid [28]. To the best of our knowledge, however, gibberellin-related *UGT* is few characterized.

The roles of *UGTs* in response to biotic and abiotic stresses have been extensively studied but their precise contribution remains elusive [29]. In *Arabidopsis*, *UGT74F1* and *UGT74F2* have been functionally characterized in their response to *Pseudomonas syringae* infection. *UGT74F2* mutant plants exhibit higher SA levels and higher levels of resistance to *Pseudomonas syringae* [30,31]. Similarly, ectopic over-expression of *UGT74F2* results in lower levels of SA and an increased susceptibility to the bacterium, while *UGT74F1* mutants exhibit lower SA levels and lowered resistance [28]. Similar results have also been reported in *UGT73B3* and *UGT73B5*, which resistant to *P. syringae* pv tomato in *Arabidopsis* [30]. There is also increasing evidence for important biological roles of *UGTs* in response to abiotic stresses. For example, overexpression of *UGT74E2* in *Arabidopsis* and *UGT85A5* in tobacco produces transgenic plants that display increased tolerance to salinity and drought stress [32,33]. Similar results have been observed in *UGT85U1/2* and *UGT85V1* in *Arabidopsis*, which have been found to be involved in salt and oxidative stress tolerance [34].

*Prunus mume*, a member of the Rosaceae family, has high ornamental value. One of striking features of *P. mume* is early flowering habit, even under relatively low temperatures in the spring [35,36]. Bud dormancy is likely responsible for this phenomenon and *UGTs* have been reported as bud dormancy candidate genes [36]. Their precise contributions have not been well defined, however, which prompted us to further explore and characterize the potential functions of *P. mume UGTs*. In the present study, we used bioinformatics techniques to carry out comparative genomic and transcriptomic analyses of *UGTs* in *P. mume* (*PmUGTs*)*.* We also analyzed the phylogenetic relationships and gene duplication history of 130 putative *PmUGTs*. Then, the expression pattern of nine group E members was tested under ABA treatment. To the best of our knowledge, this is the first report of *UGTs* on a genome-wide scale in *P. mume* and our findings should help inform future research on their potential roles in stress response.

## 2. Results

### 2.1. Identification of the Putative UGTs in P. mume

The UGT proteins play crucial roles in various plant developmental processes. Three strategies were used to identify candidate *UGT* genes in *P. mume*, as mentioned in the Material and Methods part. Subsequently, 130 potential UGT protein sequences were identified, which were named based on the chromosomal location of the corresponding genes. All these 130 putative UGT sequences started with a methionine and were full-length sequences. The protein length, molecular weight, isoelectric point and putative subcellular localization of these proteins varied widely (Supplementary Table S1). The protein sequence length and molecular weight were ranged from 279 (Pm027884) to 764 (Pm000189) amino acids (aa) and 31.25 (Pm027884) to 85.14 (Pm000189) kDa, with an average length of 470 aa and 52.45 KDa, respectively. The predicted isoelectric points varied from 4.63 (Pm000211) to 8.79 (Pm019106). Protein subcellular localization of 130 PmUGTs was also predicted by bioinformatics methods. Most of PmUGT proteins were predicted to be located in the chloroplast (75 members). 32 PmUGTs were predicted to be located in the cytoplasm and 13 were in the nucleus. More detailed information was provided in Supplementary Table S1.

### 2.2. Chromosomal Distribution, Duplication and Divergence

**Figure 1:**
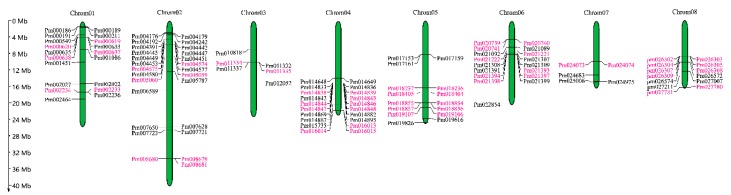
Chromosomal locations of *PmUGT* genes. The left scale represents the megabases (Mb). Chromosome numbers are shown at the top of each vertical green bar. The rough location of each maize *PmUGTs* is marked with the grey line. The tandem duplication gene pairs are highlighted with red.

**Figure 2:**
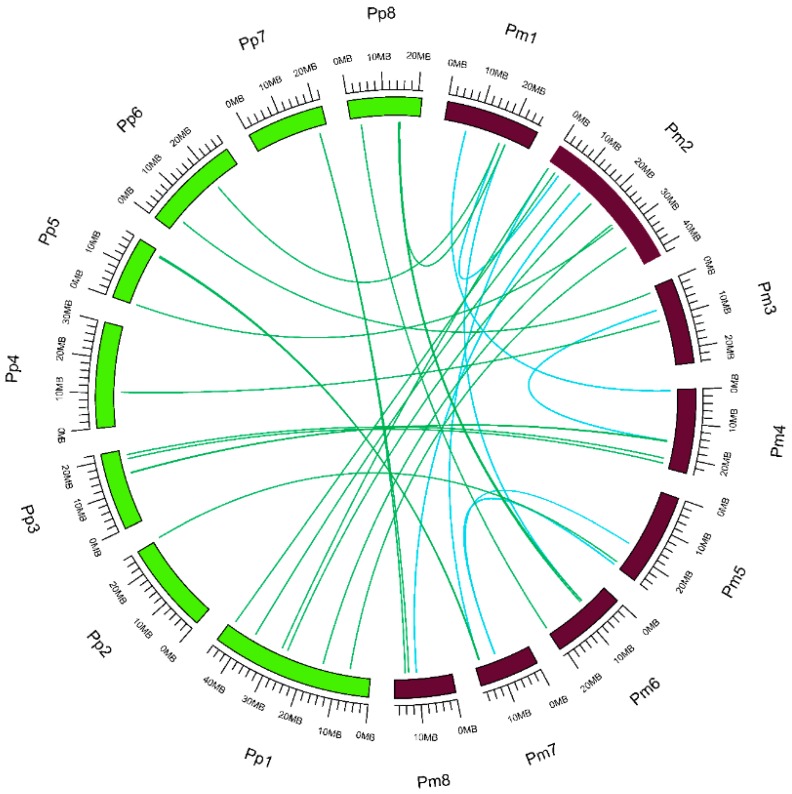
Syntenic relationships among *UGTs* in *P. mume* and *P. persica*. Chromosome are shown in the outer circle, with Pm1–8 and Pp1–8 indicated in brown and green, respectively. Genome-wide duplicated *UGTs* in *P. mume* are connected by blue lines. Genome-wide duplicated *UGTs* between *P. mume* and *P. persica* are connected by green lines.

The genomic distribution of 130 *PmUGTs* revealed that 121 *PmUGTs* distributed across eight chromosomes and nine located on scaffolds (Figure 1). There were 26 *UGTs* on chromosome 2, followed by 20, 19 and 17 members on chromosome 4, 1 and 6, respectively. Tandem and segmental duplication events were also analyzed for its importance to elucidate the chromosomal/gene segments and tandem exons. As shown in Figure 1, 32 gene pairs, including 56 *PmUGTs*, involved in tandem duplication. Moreover, eight gene pairs (*Pm001086/Pm030144*, *Pm002233/Pm004391*, *Pm002464/Pm021221*, *Pm005787/Pm026572*, *Pm010818/Pm014833*, *Pm018404/Pm024073*, *Pm019826/Pm024073*, *Pm025006/Pm030552*) were involved in the segmental duplication events (Figure 1, Supplementary Table S2). These results suggest that tandem duplication might play major roles in the *PmUGT* family amplification. When compared with *P. persica* genome, 23 segmental duplications pairs were found, as detailed in Table 1. To further analyze the syntenic relationships of *UGTs* between *P. mume* and *P. persica*, we mapped the 23 segmental duplication pairs to the duplicated blocks (Figure 2).

All the segmental duplicated UGT gene pairs had undergone a whole-genome duplication and the Ka/Ks ratios were less than 1. This result indicated that these *UGTs* experienced negative selection during species evolution process. Moreover, the divergence times of the duplicated *UGTs* at *P. mume* were significantly larger than that between *P. mume* and *P. persica*. The divergence time of the eight duplicated pairs at *P. mume* genome spanned from 47.84 (*Pm025006*-*Pm030552*) to 99.11 (*Pm005787-Pm026572*) million years ago (MYA). However, the largest divergence time of the duplicated *UGTs* between *P. mume* and *P. persica* was 72.16 MYA (*Pm021307-ppa005517 m*), followed by 56.04 MYA (*Pm002464-ppa005187 m*). Most duplicated gene pairs diverged around 1 to 5 MYA.

### 2.3. Phylogenetic Analysis of P. mume

**Figure 3:**
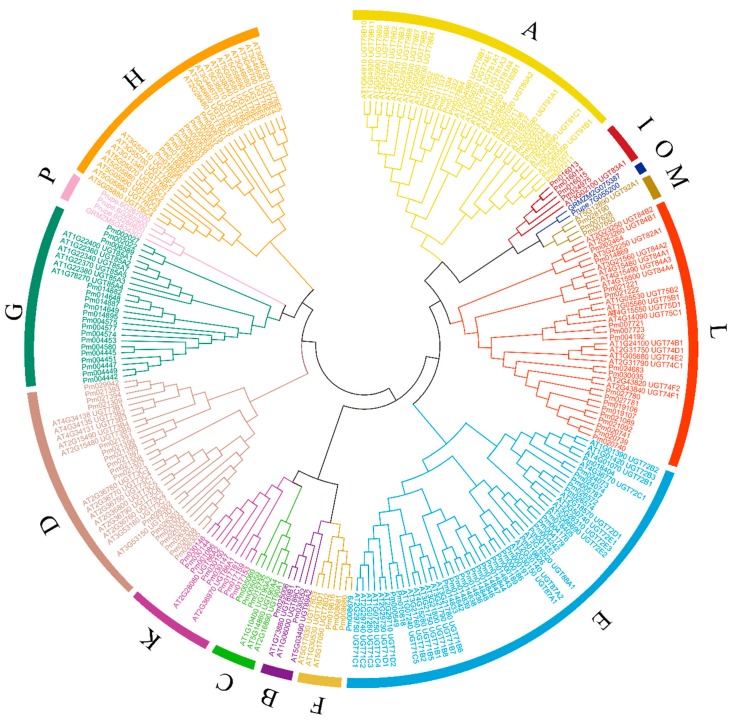
Phylogenetic tree of the plant UGTs. 130 PmUGTs, 112 *A. thaliana* UGTs, 2 maize UGTs (GRMZM2G075387 and GRMZM5G834303) and 4 peach UGTs (Prupe.7G055200, Prupe.6G265900, Prupe.6G267000 and Prupe.6G266600) were included. The full-length sequences of the UTG proteins were aligned using CLUSTALW and the phylogenetic tree was constructed using the ML method in the MEGA 6.0 [37]. The colored lines mark the groups of the UGTs.

These 130 putative *PmUGTs* and 112 *A. thaliana UGTs* (*AtUGTs*) were used for phylogenetic analysis to highlight the gene loss and gene gain events. Besides, 2 maize *UGTs* (*GRMZM2G075387* and *GRMZM5G834303*) and 4 peach *UGTs* (*Prupe.7G055200*, *Prupe.6G265900*, *Prupe.6G267000* and *Prupe.6G266600*) represented O and P groups were also added to identify *PmUGTs* O and P candidates. Phylogenetic result revealed that 14 groups, A to N, were clustered and no member was identified in group O and P (Figure 3). In each group, most of the *UGT* members were the same between *P. mume* and *A. thaliana* except in G (18 in *P. mume* and 6 in *A. thaliana*) and H (10 in *P. mume* and 19 in *A. thaliana*). Five of them possessed most of the members, with 23, 18, 17, 17 and 16 members in E, G, D, L and A groups, respectively.

### 2.4. Genomic Characteristics of the Putative UGTs in P. mume

**Figure 4:**
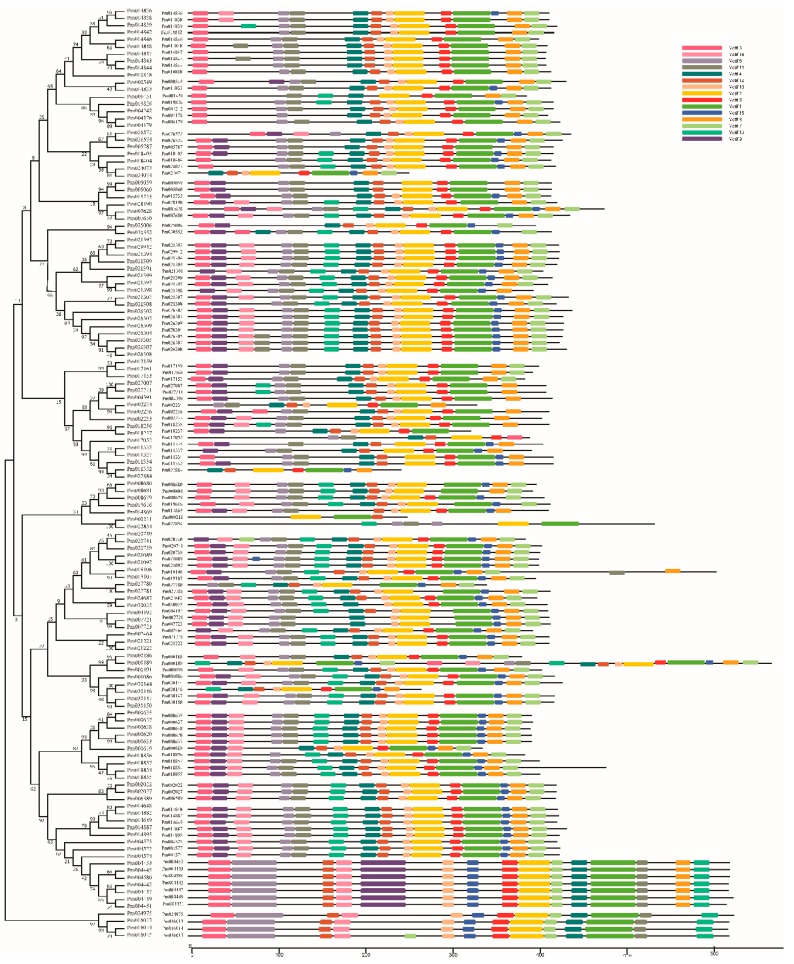
Motif distribution in *PmUGTs*. Motifs were analyzed using the MEME web server. The motifs are represented by different colors.

We analyzed the exon/intron and conserved motif characteristics of the 130 *PmUGTs* to investigate their structural diversity. Among them, 70 *UGTs* possess at least 1 intron and 60 possess no introns. Of the 70 intron-containing *UGTs*, most *UGTs* had 1–4 introns, with a ratio of 1.44 introns per intron-containing *UGTs*. And *Pm022854* contained the maximum number of introns (14), followed by *Pm000211* with 7 introns. In each phylogenetic group, the intron numbers were different. The maximum number of introns was found in E, D, L and H, whereas the minimum number of introns was found in B, C, F, I, M and N groups. It is interesting that members within each group exhibited similarity intron/extron genomic characteristics (Supplementary Figure S1). The same result was also obtained in conserved motifs structure (Figure 4). These results suggested that *PmUGT* family members within group were relatively conserved and diverged greatly among different groups.

### 2.5. Transcriptome Analysis of Tissue-Specific Expression of PmUGTs

**Figure 5:**
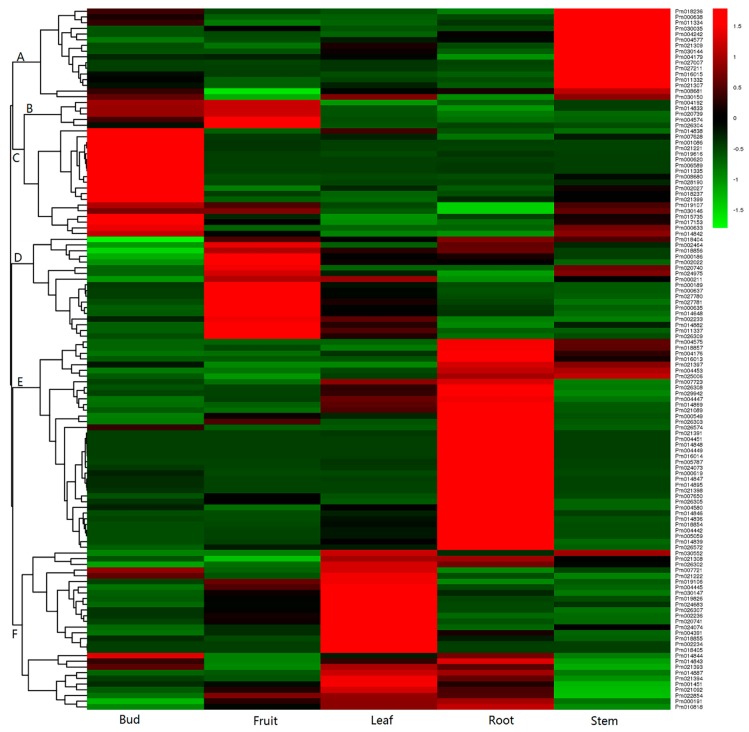
Expression profiles of *PmUGTs* in different tissues. The transcript abundances of *PmUGTs* in bud, fruit, leaf, root and stem were according to RNA-seq data. The scale represents signal intensity of FPKM values. Red indicates high relative gene expression and green indicates low relative gene expression. Letters assigned to major clusters are indicated on the dendrogram.

To detect the expression differences of *PmUGTs*, we analyzed their transcript abundances in bud, fruit, leaf, root and stem according to RNA-seq data. After filtering the low and missing expression values, 123 *PmUGTs* were finally examined to be expressed across the different tissues. Through hierarchical clustering analysis, these 123 *PmUGTs* were grouped into five discrete clusters in the five tested tissues (Figure 5). The expressed 16 *PmUGTs* in cluster A showed consistent downregulated expression patterns in bud, fruit, leaf and root but upregulated patterns in stem. The expression levels of 24 *PmUGTs* in cluster B were relatively higher in bud and fruit when compared with other tissues. In cluster C, 18 *UGTs* were detected to display upregulated expression level only in bud, with relatively low levels in other four tissues. *PmUGTs* in Cluster D, with the largest number of 37, exhibited upregulated expression in fruit; while 28 *PmUGTs* in Cluster E displayed upregulated expression in root and Cluster F showed high level in leaf (Figure 5).

### 2.6. Transcriptome Analysis of PmUGTs Expression during Bud Dormancy Transition

**Figure 6:**
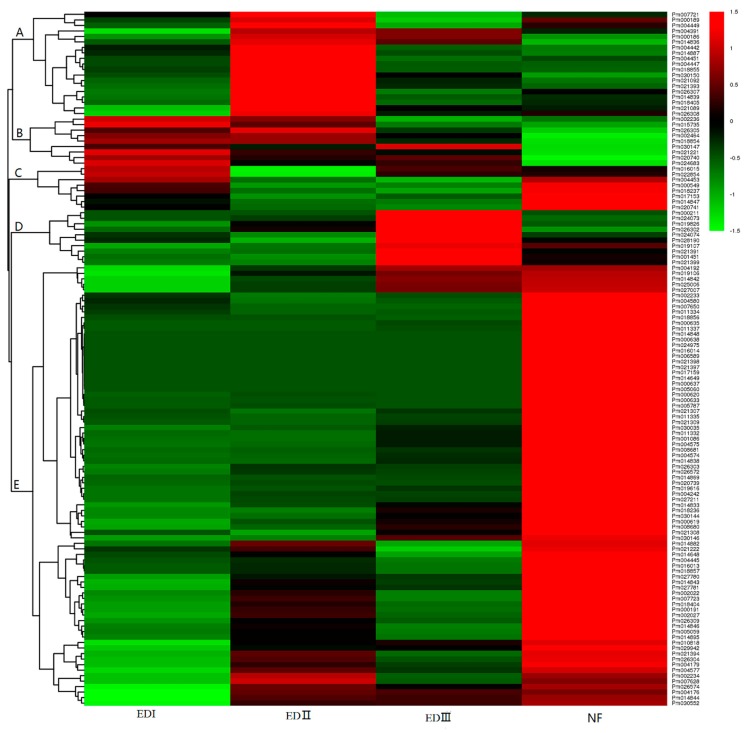
Expression profiles of *PmUGTs* at different dormancy stages. The scale represents signal intensity of FPKM values. EDI, EDII, EDIII and NF represented four dormancy stages. Red indicates high relative gene expression and green indicates low relative gene expression. Letters assigned to major clusters are indicated on the dendrogram.

In the present paper, we also displayed the *PmUGTs* expression profiles at four dormancy stages: EDI (with no flush sign in the phytotron), EDII (with 45% flush rate), EDIII (with 95% flush rate) and NF (natural flush). More details can be seen in Zhang et al. [37]. The expression profiles of *PmUGTs* during bud dormancy transition were hierarchically clustered into five groups (Figure 6). Cluster A (including 19 *PmUGTs*) exhibited highest levels at EDII and then gradually decreased as dormancy release progressed. Nine genes in Cluster B had highest level at EDI stage and then maintained relatively lower level at EDII, EDIII and NF stages. Cluster C genes showed highest expression level at EDI and sharply decreased at EDII and EDIII. After dormancy released, these genes subsequently increased at NF stage. The 10 *PmUGTs* in cluster D displayed relatively low expression levels at the EDI and EDII stages and then increased sharply at EDIII and maintained relatively high level at NF stage. Cluster E contained the large number of *PmUGTs* (80 genes). These genes showed relatively low expression level at dormancy stages and sharply increased once the dormancy completely released.

### 2.7. Quantitative Real-Time PCR Analyses of PmUGTs in Response to ABA Treatment

**Figure 7:**
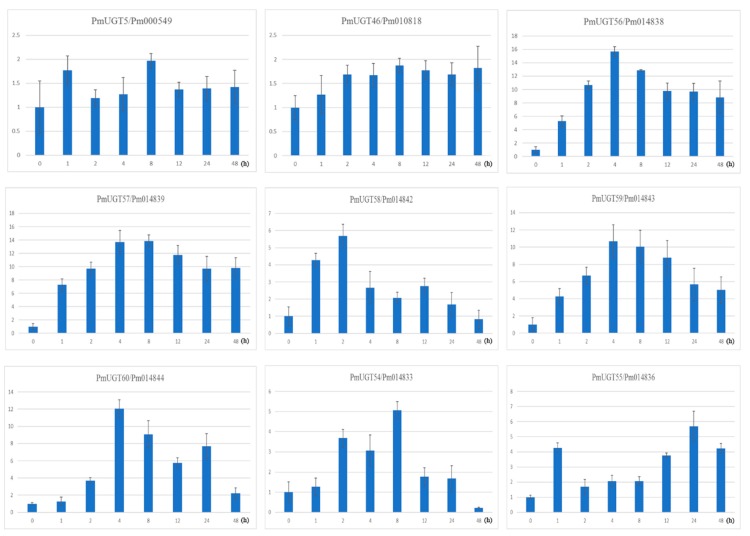
Expression patterns of *PmUGTs* in response to ABA treatment. The relative expression level of nine group E genes was examined by the RT-qPCR and normalized with the reference gene PP2A. Relative expression of 9 genes under ABA treatment at 0, 1, 2, 4, 8, 12, 24 and 48 h. The error bars represent standard deviations, *y*-axis are scales of relative expression level and *x*-axis are the time course (h) of ABA treatment.

Previous studies revealed that *UGTs* from group E might participate in response to ABA stress. In this paper, 12 group E *PmUGTs* were identified and then RT-qPCR was employed to investigate the expression patterns under ABA treatment. According to the RNA-seq data, *PmUGT61/Pm014846*, *PmUGT62/Pm014847* and *PmUGT63/Pm014848* showed extremely low expression levels in bud, leaf, stem and fruit. Moreover, these three *PmUGTs* displayed low expression pattern and showed no change during bud dormancy process. Thereafter, in this study, we only investigated the expression patterns of the other nine *PmUGTs* under ABA treatment in leaves. Of the nine *PmUGT* genes, seven genes were obviously up-regulated in response to ABA stress, while the remaining two genes *PmUGT5/Pm000549* and *PmUGT46/Pm010818* showed slight expression changes (<2-fold) (Figure 7). It is interesting that all seven selected *PmUGTs* were up-regulated at early stages and then down regulated after reaching a peak expression. The *PmUGT56/Pm014838*, *PmUGT57/Pm014839*, *PmUGT59/Pm014843* and *PmUGT60/Pm014844* were strongly up-regulated at 4 h after ABA treatment by more than 10-fold compared to the control, whereas, their expression revealed relatively decreased thereafter. Besides *PmUGT58/Pm014842*, *PmUGT54/Pm014833* and *PmUGT55/Pm014836* were slightly up-regulated by about 5-fold compared to the control. It is noting that these three *PmUGTs* showed different peaking time, suggesting their roles might be slightly different (Figure 7).

## 3. Discussion

Increasing studies indicate that UGT proteins play important roles in plant growth and adaptation to environmental stress. In addition, UGT is involved in carbohydrate metabolism during the bud dormancy release process [36]. To our knowledge, no further information is available about the UGT gene family in *P. mume*. Here, we conducted a comprehensive investigation of the *PmUGT* family.

Plant genomes contain numerous UGT genes, with *UGT* members varying among species. For example, 107 UGTs have been identified in *A*. *thaliana* [9] and 191 in *P. trichocarpa* [19]*.* Members of the UGT multigene family have also been recently identified in peach (168), grape (184), kiwifruit (188), strawberry and apple (254) [12,13,38,39,40]. In the present study, 130 UGTs were uncovered in *P. mume*, all containing the conserved PSPG box.

Phylogenetic analysis consistently clustered 14 distinct groups (A–N) with *Arabidopsis* [9]*.* This result indicates that the *UGT* family in *P. mume* has not phylogenetically diversified after separation from *Arabidopsis.* In some species of Rosaceae, including peach, apple and grape, 16 distinct phylogenetic groups (A–P) are known, while 17 groups (A–Q) have been observed in *Z. mays* [10,12,13,38]. The O and P groups found in peach and maize are absent in *P. mume*, which suggests they were lost at some stage during evolution. Surprisingly, *PmUGT* members in different groups, except for those in G and H, were similar to members of corresponding groups in *Arabidopsis*, which suggests that they have a conserved substrate specificity. Groups E, G and H were reduced in *P. mume* relative to peach, which indicates that these *UGTs* may be less critical in *P. mume*.

*PmUGTs* exhibit tissue-specific expression patterns. Determining whether the expressed UGT genes are functionally diverged or conserved should improve our understanding of plant adaptation to changing environments [4]. *PmUGT2*, *PmUGT42*, *PmUGT77*, *PmUGT80*, *PmUGT98*, *PmUGT105*, *PmUGT120* and *PmUGT121* were expressed at relatively high levels in all tested tissues, suggesting their involvement in overall tissue development process. *PmUGT17*, *PmUGT28*, *PmUGT36*, *PmUGT43*, *PmUGT49*, *PmUGT50*, *PmUGT51*, *PmUGT53*, *PmUGT73*, *PmUGT74* and *PmUGT122* were expressed at extremely low or undetectable levels in all tissues, which suggests that these genes do not play an important role in *P. mume* development. *PmUGT120* and *PmUGT32* was specifically highly expressed in leaf and in root, which implies that these genes may have a specific function in leaf and root, respectively. The same result was observed in peach. *Prupe.1G091100* and *Prupe.1G091000* (homologs of *Pm027780* and *Pm019616*, respectively) were mainly expressed in peach flowers [41]. These two *UGTs* are responsible for anthocyanin synthesis in peach flowers [41]. The dynamic expression patterns of several hormone-related *UGTs*, such as *Pm014836* (*UGT71B6*, associated with ABA), *Pm030035* (*UGT74B1*, IAA), *Pm014886* (*UGT85A1*, CK) and *Pm026307* (*UGT73C1*, CK), suggest that hormone conjugation plays important roles during the *P. mume* dormancy process*.* Even closely related homologs exhibited different spatial- and tissue-specific expression patterns. For example, the *AtUGTs*, *UGT71B6*, *UGT71B7* and *UGT71B8* exhibited very high expression levels in leaves, flowers and siliques, respectively [42].

Phytohormones play crucial roles in the regulation of protective responses against biotic and abiotic stresses but the mechanism of hormone glycosylation remains poorly understood. The availability of data from *Arabidopsis* provides sufficient information about the *UGT* family and several UGT genes have been functionally characterized as the glycoconjugates of phytohormones. For example, the *AtUGTs*, *UGT75D1*, *UGT71C5* and *UGT71B6* glycosylate ABA; *UGT74B1*, *UGT74D1* and *UGT84B1*, glycosylate IAA. In addition, *UGT74F1*, *UGT73B3* and *UGT73B5* participate in SA glycosylation, while *UGT76B1* is involved in crosstalk between SA and JA [21,31,43,44,45].

ABA is an important hormone regulating plant development and adaptive responses but information regarding ABA homeostasis is limited. The fine-tuning of ABA biosynthetic and catabolic pathways is crucial for balancing cellular ABA levels [1]. Cellular ABA content is lowered via two pathways, hydroxylation and conjugation [44,45,46,47,48]. In the first pathway, cytochrome P450 monooxygenase hydroxylates ABA at the C-80 position to form unstable 80-hydroxy ABA that is converted to phaseic acid. In the second pathway, ABA and hydroxy ABA are conjugated with glucose for inactivation [23,47,48]. It is the ABA glucosyltransferase that performs the conjugation and ABA-GE is the predominant form [23]. It is reported that ABA-GE can be transported between tissues and in some tissues, conjugation is the major pathway of ABA inactivation. Meanwhile, ABA-GE provided an ABA source for subsequent hydrolysis [43,49,50].

Several ABA-related *UGTs* and their close homologs have been functionally characterized, which can inactivate ABA and lower ABA levels. For example, ABA glycosylation by *UGT71B6*, *UGT71B7* and *UGT87A2* has been well documented in *Arabidopsis*, with this function also reported for *UGT71A33* and *UGT71A35* in strawberry and *ABAGT* in *Vigna angularis* [21,22,43,44]. As inferred by the suppression of RD29Ap:LUC, *UGT71B6*, *UGT71B7* and *UGT71B8* reduce cellular ABA levels. *UGT* RNAi (triple knock-out mutant) transgenic plants are sensitive to exogenous ABA and salinity stress during seed germination and subsequent development process. In contrast, the over-expression of *UGT71B6* in an atbg1 mutant background aggravates the ABA-deficient phenotype [42]. In the present study, 12 *UGT71B6* homologs were identified and placed in group E. We examined the transcript levels of nine of these *PmUGTs* under exogenous ABA treatment. As shown in Figure 7, seven *PmUGTs* were significantly upregulated by exogenous ABA treatment, albeit at different levels. The other two *PmUGTs* were only slightly changed. This result indicates that these *UGTs* are involved in ABA glucosylation in *P. mume*.

## 4. Materials and Methods

### 4.1. Genome-Wide Identification of UGT Family Genes in P. mume

To identify the candidate UGT genes in *P. mume*, a total of 120 Arabidopsis UGT protein sequences were retrieved from CAZy (available online: http://www.cazy.org/GlycosylTransferases.html) and 168 peach UGT proteins were downloaded from Phytozome V12.1 (available online: https://phytozome.jgi.doe.gov/pz/portal.html). All these sequences were used as query to BLASTP against *P. mume* proteome with a cut-off *E*-value of 1 × 10^−10^. Subsequently, the conserved PSPG box sequence was also used as a query to BLASTP against *P. mume* proteome database. Furthermore, the Hidden Markov Model (HMM) profile of UDPGT domain (PF00201) was retrieved from Pfam 29.0 (available online: http://pfam.xfam.org/) and used to search against the *P. mume* proteome database. The amino acid sequences of candidates from these three strategies were screened by SMART (available online: http://smart.emblheidelberg.de) to remove proteins without a complete PSPG box.

### 4.2. PmUGT Genes Location and Characteristics

InterPro was used to check the validation of final UGT genes [51]. The ORF and chromosome distribution of *P. mume UGTs* was obtained from *P. mume* genome database. MapChart (v2.3) was used to visualize the chromosomal location of *PmUGTs* [52]. ExPASy (available online: http://expasy.org/) was used to estimate the isoelectric point and molecular weight. The subcellular localization of each PmUGT was analyzed using the CELLO v2.5 server (available online: http://cello.life.nctu.edu.tw/).

### 4.3. Analyses of Gene Structure and Conserved Motifs of UGT Genes

According to the general feature format file of *P. mume*, the exon-intron structures of the *PmUGTs* were obtained and graphed with the Gene Structure Display Server (GSDS: available online: http://gsds.cbi.pku.edu.ch). The conserved motifs of the putative UGT proteins were predicted by using the on-line MEME procedure with maximum 15 motifs per sequence. The sequence logo was obtained using the online Weblogo platform (available online: http://weblogo.berkeley.edu).

### 4.4. Homology Analysis and Selection Pressures of UGT Gene Pairs between P. mume and P. persica

To estimate the divergence of the putative tandem-duplicated UGT genes between *P. mume* and *P. persica*, the duplicated pairs were detected in the Plant gene duplication database (available online: http://chibba.agtec.uga.edu/duplication/). Mcscan [53] was employed to identify homologous regions and syntenic blocks were evaluated using Circos-0.64 [54]. The ratios of Ka (non-synonymous)/Ks (synonymous rate) of UGT gene pairs between *P. mume* and *P. persica* were calculated to estimate selection modes by using PAML software. 1.5 × 10^−8^ was taken as synonymous substitutions per site per year in the case of dicotyledonous plants for MYA calculation. The Ka/Ks ratios greater than 1, equal to 1 and less than 1 represent positive, neutral and negative selection, respectively.

### 4.5. Sequence Alignments, Phylogenetic Analyses of UGT Genes

The UGT protein sequences, including 130 PmUGT, 112 AtUGTs, 2 maize UGTs (GRMZM2G075387 and GRMZM5G834303) and 4 peach UGTs (Prupe.7G055200, Prupe.6G265900, Prupe.6G267000 and Prupe.6G266600) were used for phylogenetic analysis by program CLUSTALW in MEGA 6.0 software [37]. Then, the output alignment file was used to construct Maximum Likelihood (ML) trees with pair-wise deletion and 1000 replications.

### 4.6. Transcriptome Analysis for Tissue-Specific Expression

To check tissue-specific expression of the putative *UGTs* in *P. mume*, the RNA-Seq data in different tissues, such as flower, leaves, roots and stem, were obtained. Besides, the transcript data at four crucial dormancy stages were also retrieved as detailed described by Zhang et al. [36]. The expression values for each *PmUGT* were calculated by fragments per kilobase of the exon model per million mapped reads by using the RNA-seq data of *P. mume*. The heat-maps of *PmUGTs* were established using R packages “heatmap”.

### 4.7. RT-qPCR Analyses of the PmUGTs in Response to ABA Treatment

The seeds of *P. mume* were collected on cultivar “Lve” grown in the Jiufeng International Plum Blossom Garden, Beijing, China (40°07′ N, 116°11′ E). The seeds were sterilized with 20% sodium hypochlorite, washed with sterile water three times and were stored in the sand under 4 °C to promote germination. After three months, germinated seedlings were transplanted in nutritional soil in the greenhouse. For hormone treatment, 100 µM ABA were sprayed on the young seedlings until dropped. Fresh leaves were collected at 0, 1, 2, 4, 8, 12, 24 and 48 h, respectively. Samples were frozen in liquid nitrogen and then stored at −80 °C until used. Total RNA extraction and qPCR were performed as described in Zhang et al. [37] Primers sequences were listed in Supplementary Table S3.

## 5. Conclusions

A total of 130 *PmUGTs* were identified and clustered into 14 groups based on phylogenetic analysis and their chromosomal locations, gene structure, duplication events and conserved motifs were further investigated. RNA-seq analysis revealed specific expression patterns in different tissues. In addition, various changes in transcript levels were detected during bud dormancy release. We also uncovered differential responses of *PmUGT* expressions to ABA treatment using RT-qPCR. A major future research challenge is obtaining a better understanding of how plants regulate *UGT* members during development and in response to abiotic and biotic stress. Exploring the crosstalk between *UGTs* and other genes/proteins is also necessary. Our results provide important information on the UGT family in *P. mume* that will aid the further characterization of their biological roles in response to environmental stress.

## Figures and Tables

**Table 1:** Calculation of Ka/Ks and the divergence time of the duplicated UGT gene pairs in *P. mume* and *P. persica* genomes.

Duplicated Gene Pairs	Ka	Ks	Ka/Ks	Duplication Type	Purifying	Time (MYA)
Pm001086-Pm030144	0.2807	2.0011	0.140273	WGD/Segmental	Yes	66.70
Pm002233-Pm004391	0.4411	1.7464	0.252577	WGD/Segmental	Yes	58.21
Pm002464-Pm021221	0.5638	1.6179	0.348476	WGD/Segmental	Yes	53.93
Pm005787-Pm026572	0.7050	2.9734	0.237102	WGD/Segmental	Yes	99.11
Pm010818-Pm014833	0.4487	1.7679	0.253804	WGD/Segmental	Yes	58.93
Pm018404-Pm024073	0.3273	1.6211	0.2019	WGD/Segmental	Yes	54.04
Pm019826-Pm024073	0.6853	2.1848	0.313667	WGD/Segmental	Yes	72.83
Pm025006-Pm030552	0.4543	1.4351	0.316563	WGD/Segmental	Yes	47.84
Pm002233-ppa021249m	0.0534	0.2157	0.247566	WGD/Segmental	Yes	7.19
Pm002464-ppa005187m	0.5639	1.6813	0.335395	WGD/Segmental	Yes	56.04
Pm004192-ppa024612m	0.0130	0.0217	0.599078	WGD/Segmental	Yes	0.72
Pm005059-ppa017646m	0.0195	0.0815	0.239264	WGD/Segmental	Yes	2.72
Pm006589-ppa020820m	0.0124	0.0599	0.207012	WGD/Segmental	Yes	2.00
Pm007628-ppa023949m	0.0526	0.1238	0.424879	WGD/Segmental	Yes	4.13
Pm007721-ppa005161m	0.033	0.1163	0.283749	WGD/Segmental	Yes	3.88
Pm008679-ppa017941m	0.0432	0.0867	0.49827	WGD/Segmental	Yes	2.89
Pm011332-ppa005654m	0.175	0.7565	0.231328	WGD/Segmental	Yes	25.22
Pm014846-ppa023681m	0.172	0.7111	0.241879	WGD/Segmental	Yes	23.70
Pm014869-ppa016262m	0.0129	0.0481	0.268191	WGD/Segmental	Yes	1.60
Pm015735-ppa024768m	0.204	0.4454	0.458015	WGD/Segmental	Yes	14.85
Pm016014-ppa024744m	0.3188	0.8492	0.375412	WGD/Segmental	Yes	28.31
Pm019616-ppa005162m	0.015	0.0354	0.423729	WGD/Segmental	Yes	1.18
Pm021221-ppa005187m	0.0177	0.05	0.354	WGD/Segmental	Yes	1.67
Pm021307-ppa005517m	0.3865	2.1649	0.17853	WGD/Segmental	Yes	72.16
Pm022854-ppa002535m	0.0032	0.037	0.086486	WGD/Segmental	Yes	1.23
Pm024975-ppa022508m	0.0142	0.0735	0.193197	WGD/Segmental	Yes	2.45
Pm025006-ppa018626m	0.0182	0.046	0.395652	WGD/Segmental	Yes	1.53
Pm027007-ppa025742m	0.0298	0.0677	0.440177	WGD/Segmental	Yes	2.26
Pm027211-ppa025742m	0.0267	0.0889	0.300337	WGD/Segmental	Yes	2.96
Pm030552-ppa024271m	0.0187	0.0568	0.329225	WGD/Segmental	Yes	1.89
Pm028190-ppa016005m	0.0412	0.0547	0.753199	WGD/Segmental	Yes	1.82

MYA, Millions of years ago; Ks, synonymous substitutions; Ka, nonsynonymous substitutions; Ka/Ks, nonsynonymous substitutions per synonymous site.

## Supplementary Materials

Supplementary materials can be found at http://www.mdpi.com/1422-0067/19/11/3382/s1.
